# TYK2 Protein Expression and Its Potential as a Tissue-Based Biomarker for the Diagnosis of Colorectal Cancer

**DOI:** 10.3390/cancers16213665

**Published:** 2024-10-30

**Authors:** Łukasz Zadka, Adam Ustaszewski, Natalia Glatzel-Plucińska, Agnieszka Rusak, Izabela Łaczmańska, Katarzyna Ratajczak-Wielgomas, Alicja Kmiecik, Aleksandra Piotrowska, Katarzyna Haczkiewicz-Leśniak, Agnieszka Gomułkiewicz, Magdalena Kostrzewska-Poczekaj, Piotr Dzięgiel

**Affiliations:** 1 Division of Ultrastructural Research, Faculty of Medicine, Wroclaw Medical University, Chałubińskiego 6a, 50-368 Wrocław, Poland; katarzyna.haczkiewicz-lesniak@umw.edu.pl; 2 Institute of Human Genetics, Polish Academy of Sciences, 60-479 Poznań, Poland; adam.ustaszewski@igcz.poznan.pl (A.U.); magkos@man.poznan.pl (M.K.-P.); 3 Division of Histology and Embryology, Department of Human Morphology and Embryology, Faculty of Medicine, Wroclaw Medical University, 50-368 Wrocław, Poland; natalia.glatzel-plucinska@umw.edu.pl (N.G.-P.); katarzyna.ratajczak-wielgomas@umw.edu.pl (K.R.-W.); alicja.kmiecik@umw.edu.pl (A.K.); aleksandra.piotrowska@umw.edu.pl (A.P.); agnieszka.gomulkiewicz@umw.edu.pl (A.G.); piotr.dziegiel@umw.edu.pl (P.D.); 4 Department of Genetics, Faculty of Medicine, Wroclaw Medical University, 50-368 Wrocław, Poland; izabela.laczmanska@umw.edu.pl

**Keywords:** tyrosine kinase 2, colorectal cancer, CRC, immunohistochemistry

## Abstract

Background/Objectives: The aim of this study was to examine the expression of TYK2 in colorectal cancer (CRC) and to determine the potential diagnostic and prognostic significance of this kinase. Methods: Digital image analysis was performed to assess immunohistochemical TYK2 reactivity. Results: There were significant differences for all positive pixels between CRC and normal colonic mucosa, with higher TYK2 expression levels observed in surgical margins than in adenocarcinomas (*p* = 0.0004). Paired *t* tests showed elevated immunoreactivity for overall TYK2 expression in matched pairs of CRC with adjacent surgical margins (*p* < 0.0001). Higher percentages of weak (*p* < 0.0001) and strong pixels (*p* = 0.0260) were detected in normal colonic mucosa than in cancer tissues. To distinguish cancer from normal intestinal mucosa, the following cutoffs for the TYK2 immune score were found: 29.5% for all cases and 31% for matched pairs. Tumor budding (Bd) was negatively correlated with the percentage of strong pixels for TYK2 (ρ = −0.270, *p* = 0.0096). The percentage of strong pixels was significantly elevated for the T parameter (*p* = 0.0428). There was a positive correlation between the number of involved lymph nodes and weak pixels (ρ = 0.239, *p* = 0.0242). Immunofluorescence staining showed significantly higher signal intensities in colonic mucosa than in CRC. The protein level of TYK2 was significantly higher in controls than in cancer tissues. TEM imaging showed lower levels of TYK2 in cancer than in ulcerative colitis. Conclusions: TYK2 protein expression may bring diagnostic value in patients diagnosed with CRC.

## Simple Summary

The tyrosine kinase TYK2 was assessed in this study using digital image analysis of archived formalin-fixed paraffin-embedded blocks. The immunohistochemical expression of TYK2 was significantly weaker in cancer than in normal control tissues, and its high predictive value allowed its use as a biomarker in the diagnosis of CRC. The obtained results were additionally verified by other molecular methods, which confirmed the presence of a higher protein level of the kinase TYK2 and a higher TYK2 fluorescence intensity in normal colonic mucosa than in CRC tissue. Independently assessed TYK2 immunoreactivity in fresh colonic biopsies from CRC and ulcerative colitis (UC) patients showed that the kinase distribution was greater in UC than in cancer.

## 1. Introduction

Tyrosine kinase 2 (TYK2) is an important immunomodulatory factor of innate and adaptive immune responses and is considered a drug target for selected autoimmune disorders and inflammatory bowel diseases [1]. An inflamed colonic mucosa resulting from chronic inflammation is a well-known pre-cancerous lesion that predisposes patients to the development of colorectal adenocarcinoma in the long term [2]. Moreover, this kinase regulates signaling pathways that are related to the pathogenesis and treatment response of colorectal cancer (CRC); however, the impacts of these pathways on the development of this cancer seem to be divergent [1,3,4]. Despite the unknown influence of TYK2 on the mechanisms of oncogenesis in CRC, selective inhibitors of this kinase are currently undergoing clinical trials with a view to commercialization. The importance of TYK2 in the pathogeneses of numerous inflammatory conditions has also led to the development of new compounds with increased selectivity and favorable pharmacokinetic properties, driving a significant increase in the registration of new selective inhibitors [5,6,7,8,9].

Thus far, more studies have shown the beneficial effects of TYK2 on the cellular immune response and the epithelial lining of the large intestine. TYK2 regulates the maturation and cytotoxicity of natural killer (NK) cells and enhances the release of IFN-γ by this subtype of lymphocytes. Moreover, in the gastrointestinal tract, it has positive effects on epithelial homeostasis and changes occurring in the intestinal microflora [10,11]. Although there is still insufficient research assessing the importance of TYK2 in CRC, it is worth noting that two decades have passed since the tyrosine kinase’s expression profile in normal colonic mucosa was assessed at the mRNA level and compared with that in various stages of carcinogenesis toward CRC. At that time, the TYK2 mRNA transcript was detected only in colorectal polyps, with no trace of its expression in cancer. Moreover, this archival study was performed on a limited number of patients, and TYK2 expression at the protein level was not estimated [12]. In this study, we show for the first time that the expression level of TYK2 in colorectal adenocarcinomas significantly differs from that in normal colonic mucosa samples that has diagnostic value and is associated with clinicopathological data. These findings suggest that TYK2 is the first known tissue biomarker for CRC.

## 2. Materials and Methods

### 2.1. The Patients and Samples

The study was conducted on archived formalin-fixed, paraffin-embedded (FFPE) blocks and the corresponding frozen tissues stored at the Department of Human Morphology and Embryology at Wroclaw Medical University. The archived tissue material was collected in 2012–2016 from patients undergoing colectomy. The tumor masses and corresponding healthy margins were removed during surgical procedures and immediately processed under complete standardization. Whenever samples were taken, fixation was performed in buffered formalin and the time of fixation did not exceed 48 h prior to further tissue processing. Additionally, fresh tissue material was collected in 2020 from endoscopic biopsies of patients who underwent colonoscopy and were diagnosed with de novo CRC and ulcerative colitis at the Gastroenterology Department of the 4th Military Hospital in Wroclaw. A histopathological diagnosis was made by a clinical pathologist. None of the patients enrolled in this study underwent preoperative radio- and/or chemotherapy. Lack of tissue material due to use of the specimens was the criterion for excluding an individual case from further research procedures. Immunohistochemical staining was ultimately performed on 88 patients with a histological diagnosis of primary colorectal adenocarcinoma. In 27 cases of FFPE blocks, comparative IHC against TYK2 was performed in surgical margins, which were defined as normal controls. The absence of neoplastic infiltrations in the margins was verified by standard H&E staining (ŁZ, PD). The study protocol was approved by the Bioethics Committee at Wroclaw Medical University (protocol number: 609/2021). The clinicopathological data of the archived samples are presented in Table 1. The characteristics of the clinical cohort tested by transmission electron microscopy as well as patient recruitment criteria and sample handling procedures are described in detail in the TEM section.

### 2.2. Tissue Microarrays (TMAs)

TMAs were performed with the use of a TMA Grand Master (3DHistech, Budapest, Hungary) automatic tissue microarrayer. From each FFPE block, 6 μm thick sections were cut and stained with H&E. Next, the prepared slides were scanned with a Pannoramic MIDI II (3DHistech) histological scanner. Three representative tissue cores that were 1.5 mm in diameter each were extracted from selected FFPE blocks (AP). Each core was selected from the digital slides using CaseViewer 2.4 version software (3DHistech). The selection of the most representative cores for the TMA procedure was supported by the expertise of a qualified pathologist (PD).

### 2.3. Immunohistochemistry (IHC)

IHC was performed on 8 μm thick slides from FFPE blocks (AP). Initially, the slides were deparaffinized, and endogenous peroxidase was blocked for 10 min with Envision Flex Peroxidase-Blocking Reagent (Agilent, Santa Clara, CA, USA). Rabbit polyclonal antibodies against TYK2 (Abcam (Cambridge, UK), ab223733) were used as primary antibodies (20 min). According to the manufacturer’s instructions, the purchased antibodies were validated using a knockout cell line. Then, the slides were incubated in EnVision FLEX/HRP (20 min). The reactions were visualized with freshly prepared 3,3′-diaminobenzidine (DAB) chromogen (5 min). Histological slides were stained additionally with EnVision Flex hematoxylin (5 min). After IHC and staining, the slides were dehydrated in ethanol (70%, 96%, absolute) and xylene and then sealed with Dako Mounting Medium (Agilent). Further reactions were performed on a Dako Autostainer Link48 (Agilent).

### 2.4. Computational Assessment of Digital Slides of TMA Cores

**Figure 1:**
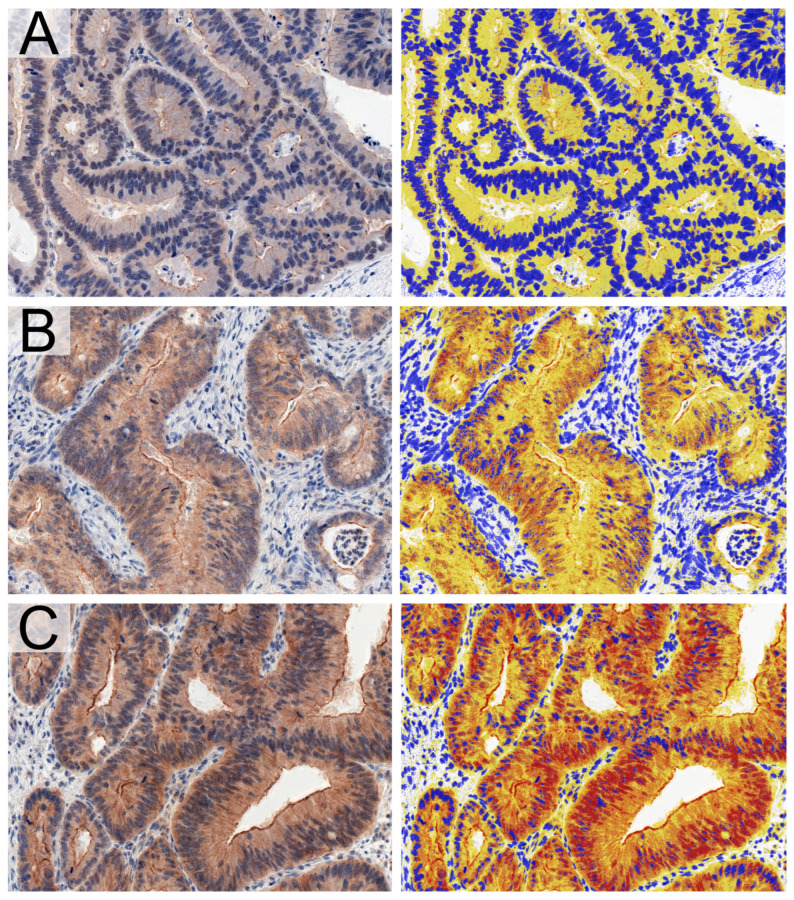
TYK2 immunohistochemical staining in colorectal adenocarcinomas (left) and its counterparts in digital image analysis showed positive reactions of weak (**A**), moderate (**B**) and strong (**C**) intensity; 20× magnification.

Colorectal cancer and matched cancer-associated normal colonic mucosa tissues with representative immunohistochemical staining of expressed TYK2 were assessed using a digital method with QuantCenter 2.2 software (3DHistech). For this purpose, each histological slide was scanned using a Pannoramic MIDI II scanner (3DHistech) at 40× magnification, and the resulting images were preprocessed for further evaluation with CaseViewer (3DHistech). To determine the percentage of cells displaying positive TYK2 expression, the DensitoQuant (3DHistech) software (2.2 version) module was used (NGP). The estimation was appropriate for tumor cells or normal epithelial cells only; therefore, inflammatory cells and tumor stroma were excluded from the digital analysis. The obtained results are presented as the percentage of positive pixels with the exact immunohistochemical staining intensity divided into weak (w), moderate (m) and strong (s) categories (Figure 1).

### 2.5. Whole Slide Imaging Using Confocal Microscopy

For immunofluorescence (IF) staining, FFPE blocks were cut into 4 μm thick sections. Deparaffinization, hydration and thermal epitope retrieval were performed using a low-pH Target Retrieval Solution (Agilent Technologies, Santa Clara, CA, USA) for 20 min at 97 °C on a Dako PT Link module (Dako, Glostrup, Denmark). Sites of nonspecific binding were blocked using 3% BSA in PBS (1 h/RT). The slices were incubated at 4 °C overnight with primary anti-TYK2 antibodies (1:200 dilution; Abcam, Cambridge, UK, ab223733) in 3% BSA/PBS. Next, the whole slides were rinsed with PBS and incubated for 1 h with donkey anti-rabbit secondary Alexa Fluor 568 conjugated antibodies (dilution 1:2000, Abcam, Cambridge, UK, ab175470, RRID: AB_2783823). Negative controls were treated with 1% BSA in PBS instead of the specific antibody (AK). The preparations were mounted in Prolong DAPI Mounting Medium (Invitrogen, Thermo Fisher Scientific, Waltham, MA, USA). Observations were made at 600× magnification (KRW) using a Fluoview FV3000 confocal microscope (Olympus, Shinjuku, Tokyo, Japan) coupled with the Cell Sense software (Version 3.2) (Olympus, RRID:SCR_016238, Shinjuku, Tokyo, Japan).

### 2.6. Immunoblotting

Immunoblotting was performed to examine the protein level of TYK2. Isolation of protein from frozen tissue samples was carried out with a TissueRuptor homogenizer (Qiagen, Hilden, Germany) and the T-PER Tissue Protein Extraction Reagent with 0.5 mM PMSF (phenylmethanesulfonyl fluoride), EDTA (ethylenediaminetetraacetic acid) and protease inhibitor (Heat Protease Inhibitor Cocktail × 100) (Thermo Scientific, Wilmington, DE, USA). A bicinchoninic acid and colorimetric assay kit (Pierce BCA Protein Assay Kit) and NanoDrop 1000 spectrophotometer (Thermo Scientific) were used to determine the protein level. Denaturation of tissue lysates was conducted in a buffer solution containing 250 mM TRIS pH 6.8, 40% glycerol, 20% (*v*/*v*) β-mercaptoethanol, 0.33 mg/mL bromophenol blue and 8% sodium dodecyl sulfate (SDS) for 10 min at 95 °C. SDS–PAGE protein separation in an 8% polyacrylamide gel (Mini Protean 3 apparatus, Bio-Rad, Marnes-la-Coquette, France) was performed with 0.1 mg of protein per lane. Protein transfer was performed in Tris-glycine buffer with 10% methanol and 0.1% SDS on a nitrocellulose membrane with a 0.45 μm pore size (Whatman Protran, GE Healthcare Life Sciences, Marlborough, MA, USA) for 2 h at 100 V. The membranes were blocked in 2% BSA in 0.1% TBST and incubation with a primary antibody directed against TYK-2 (1:1000, ab223733, Abcam, Cambridge, UK) was carried out overnight at 4 °C. Next, the membranes were washed three times in 0.1% TBST and incubated with secondary donkey-anti rabbit antibodies conjugated with horseradish peroxidase (HRP) (1:3000, in 0.1% TBST, 1 h, RT, Jackson ImmunoResearch, Suffolk, UK). After blocking with 5% milk in 0.1% TBST (1 h, RT) incubation with a primary antibody directed against the reference protein β-actin (1:2000, in 1% milk in 0.1% TBST, MA5-15739, Thermo Scientific) was carried out overnight at 4º C and was followed by incubation with secondary donkey-anti mouse antibodies conjugated with HRP for 1 h at RT (1: 3000, in 0.1% TBST, Jackson ImmunoResearch). The level of TYK-2 was determined by semi-quantitative densitometric analysis and normalized to the β-actin level. The chemiluminescence reaction was detected with Immobilon Forte Western HRP substrate (Millipore, Bedford, MA, USA) and a ChemiDoc MP System (Bio-Rad, Hercules, CA, USA) with an exposure time from 6 s to 2 min using ImageLab 6.1 software (Bio-Rad). The experiments were run in triplicates (AR).

### 2.7. DNA Isolation from FFPE Blocks

For the selected FFPE blocks, a complete set of clinical and pathological data was available. For each patient there was a pair of matched FFPE blocks representing tissue fragments collected during the surgical removal of primary adenocarcinomas and their corresponding normal colonic mucosa tissues of the large intestine obtained from the healthy surgical margins. The microanatomy of the surgical margins constituting the normal controls was additionally verified on the performed histological slides with standard H&E staining to exclude the presence of any cancer cells (ŁZ, PD). All samples were collected in 2012–2016 under complete standardization. From each paraffin block, 6 sections of 5 µm thickness were shaved and collected in 2 mL clean tubes. The first two sections of tissue were rejected as suggested in the manufacturer’s instructions. For DNA purification, a QIAamp DNA FFPE Tissue Kit (Qiagen, Hilden, Germany) was used according to the manufacturer’s protocol with minor modifications. First, 30 µL of Proteinase K solution was added to the pellet suspended in 180 µL of ATL buffer, and the incubation time at 56 °C was extended to 2 h. To remove residual droplets from the center of the lid of each tube, the samples were centrifuged briefly for 16 s at 24 °C and with a spin speed of 6000× *g* (Eppendorf 5417R, Hamburg, Germany). The recovered lysates in a volume of 600 µL per sample were transferred to QIAamp MinElute columns. Then, 25 μL of ultrapure H_2_O (Sigma-Aldrich (Burlington, MA, USA)) was applied to the QIAamp MinElute columns instead of the ATE buffer. The purified DNA was transferred to new 1.5 mL Eppendorf tubes and analyzed for quality and concentration using a NanoDrop (ND-1000) spectrophotometer, where an A260/280 ratio ≥ 1.8 was considered to be sufficient (ŁZ). The samples were stored at −20 °C.

### 2.8. DNA Bisulfite Pyrosequencing

Purified DNA (~500 ng per sample) was used for further procedures. The DNA was bisulfite-converted using an EZ DNA Methylation-Gold Kit (Zymo Research, Irvine, CA, USA) according to the manufacturer’s protocol. PCR mixtures were prepared in a UVC/T-AR DNA/RNA UV-cleaner box (Biosan, Riga, Latvia) using a PyroMark PCR Kit (Qiagen, Hilden, Germany). The reaction mixture contained 12.5 µL of PyroMark Master Mix, 0.5 µL (20 pmol/µL) of F and R primer, 2.4 µL of CoralLoad, 1 µL of converted DNA (~25 ng/µL) and 8 µL of RNase-Free water. PCR was performed under the following conditions: 95 °C for 15 min × 1; 94 °C for 30 s, 59 °C for 30 s, 72 °C for 30 s × 45; 72 °C for 10 min × 1; and 4 °C ∞. The PCR products were visualized on a 1.8% agarose gel with SimplySafe (EURx, Gdansk, Poland; cat. no. E4600) under UV light (BioDoc-it Imaging System, UVP, USA). Further purification of the PCR products followed by bisulfite pyrosequencing was performed using a PyroMark Q24 sequencer as described previously (Qiagen, Hilden, Germany) [13].

For analysis of TYK2 promoter region methylation, a bisulfite pyrosequencing assay was designed using PyroMark Assay Design 2.0 software (Qiagen). The following PCR primers were used: F: 5′ GGATGGGAGTATTTGGTGTT 3′ and R: 5′ ATATCCTCCCCCCAAAACCTCTAAA 3′ (Seq: 5′ GTGGGTGTATATTTTAGTTT 3′). This assay was used for bisulfite sequencing of the following DNA fragment: chr19:10,380,342–10,380,359 GRCh38/hg38. The fragment included four CG dinucleotides (chr19:10,380,345, chr19:10,380,349, chr19:10,380,355 and chr19:10,380,358) used for the analysis of DNA methylation of the TYK2 promoter region. Each pyrosequencing experiment included a fully methylated DNA control (CpG Genome Universal Millipore, Darmstadt, Germany) and an unmethylated whole genome amplified (WGA) control obtained using a GenomePlex Whole Genome Amplification Kit (Sigma-Aldrich, Steinheim, Germany).

### 2.9. DNA Methylation of TYK2 in Matched Primary Colorectal Adenocarcinomas and Normal Colonic Mucosa Samples

We analyzed the TYK2 promoter region DNA methylation level as a potential mechanism responsible for the downregulation of this gene in cancer cells. The four CG nucleotides localized in the potential TYK2 promoter were tested in primary colorectal adenocarcinoma and normal colonic mucosa samples. No significant changes in DNA methylation were observed in tumor samples in comparison to the controls (Table 2).

### 2.10. Fluorescence In Situ Hybridization (FISH)

TYK2-specific labeled FISH probes were used to detect amplifications and deletions associated with this gene. FISH analysis was performed using a TYK2 (19p13.2)-specific probe (gold) (Empire Genomics) along with a chromosome 16 centromeric enumeration probe (CEP16) (green) (Empire Genomics). Cancer and adjacent normal tissues from a single donor were examined. All diagnostic procedures were performed according to the manufacturer’s protocols. FFPE blocks were sectioned at 4 µm thickness, staged at 90 °C for 25 min, deparaffinized using fresh xylene for 10 min and immersed in 98.9% ethanol for 5 min. The slide pretreatment steps included incubation at 95 °C in citric acid pretreatment buffer (pH ~6.8) for 30 min, washing in 2xSSC for 5 min and drying. For 100 µL of digestion pepsin solution, Digest-ALL (Life Technologies (Carlsbad, CA, USA)) was applied to the tissue fragment (40 min, 37 °C) and the slides were washed in 2xSSC for 5 min and immersed in 70% ethanol for 30 s. On dried slides, a 10 μL probe mix per cellular area (2 μL of TYK2 probe and 2 μL of CEP16 probe with 6 μL of hybridization buffer) was applied. The slides were denatured at 75° C for 10 min and hybridized at 37 °C for 16–24 h. The post-hybridization wash steps included a wash in 0.3% Igepal (Sigma-Aldrich)/0.4xSSC (Applichem) at 73 °C for 2 min and a wash in 0.1% Igepal/2xSSC at room temperature (RT) for 2 min. DAPI with antifade solution (Vysis) was applied to the dried slides. The fluorescent signals were detected for 100 nuclei on each hybridization area using an Imager.M1 (Zeiss) microscope and Isis software (6.3 version) (Metasystems DE).

### 2.11. Real-Time Quantitative Polymerase Chain Reaction (Real-Time PCR)

To assess the degree of gene expression for TYK2, quantitative evaluation of mRNA by real-time PCR was performed. The assessed solid tissue material consisted of 32 frozen samples, including 28 samples representing matched pairs of CRC and corresponding normal colonic mucosa tissues (n = 14 patients) secured during surgical removal of the tumor mass and 4 samples resected from the large intestine during a colonoscopy examination (two primary colonic adenocarcinomas and two cases of ulcerative colitis). All samples were fixed in RNAlater immediately after biopsy. The tissue material obtained from these two patient cohorts was assessed separately. Prior to RNA isolation, all samples were homogenized using a TissueRuptor homogenizer (Qiagen, Hilden, Germany). Total RNA was extracted using a RNeasy Mini Kit (Qiagen) following the manufacturer’s instructions (ŁZ, AR). To eliminate genomic DNA contamination, on-column DNase digestion was performed using RNase-Free DNase Set (Qiagen). Assessment of RNA quality and integrity was performed by capillary electrophoresis using an Agilent 2100 bioanalyzer and an associated RNA 6000 Nano LabChip Kit (Agilent Technologies, Palo Alto, CA, USA). Next, the total RNA was transcribed into single-strand cDNA with a High-Capacity cDNA Reverse Transcription Kit (Applied Biosystems, Foster City, CA, USA). The relative TYK2 mRNA expression levels were determined using a 7500 Real-Time PCR System (Applied Biosystems, Foster City, CA, USA). The sets of primers and TaqMan probes used in this study were as follows: Hs01105953_m1 was used for TYK2 and Hs99999903_m1 was used for the reference gene ACTB (Applied Biosystems). The reactions were performed with the following settings: activation of polymerase at 50 °C for 2 min, initial denaturation at 94 °C for 10 min followed by 40 cycles of denaturation at 94 °C for 15 s and annealing and elongation at 60 °C for 1 min. The analysis was carried out using the ΔΔCt method in both groups, with reference to the samples with the lowest relative mRNA expression level (AG).

### 2.12. Transmission Electron Microscopy (TEM)—Tissue Processing

TEM analysis was performed to detect and quantify the TYK2 kinase distribution at the subcellular level.

#### 2.12.1. Solid Tissue Collection and Fixation

Four colonic biopsies were taken directly from patients who underwent colonoscopy procedures in the Gastroenterology Department at the 4th Military Teaching Hospital in Wroclaw, Poland. Tissues were collected only from sites affected by disease that were found incidentally and from de novo patients without prior diagnosis. The fresh samples were immediately embedded in cooled 4% formaldehyde solution (FA) (1 mL of 16% formaldehyde solution [*w*/*v*], methanol-free (Thermo Fisher Scientific, Waltham, MA, USA) and 3 mL of phosphate buffer saline [PBS, pH 7.4]) and transported on ice directly to the Department of Human Morphology and Embryology, Histology and Embryology Division, Wroclaw Medical University, Poland within 35 min. Next, the samples were stored in FA for 24 h at 4 °C. Then, the FA was rinsed away 3 times for 5 min with PBS at room temperature (RT). The patients were selected for this study based on histopathological diagnosis and clinicopathological data. Two patients were diagnosed with ulcerative colitis based on histopathological diagnosis and the symptoms of the disease. Considering the anatomical locations of the primary lesions, rectal biopsies were taken from those patients (male, 62 years; female, 78 years). From the two other patients with endoscopically diagnosed intestinal tumor masses, fresh colonic biopsies were taken from the cecum (female, 85 years) and ascending colon (male, 84 years). After the histopathological examination, a clinical diagnosis of colonic adenocarcinoma was made in both cases (G3 and G1 grades).

#### 2.12.2. Embedding in LR White Resin

The specimens were dehydrated in increasing concentrations of ethanol, EtOH (50%, 70%, 96%, 99,8% [Stanlab, Lublin, Poland]) at RT, and each incubation with EtOH lasted 10 min. Afterward, the samples were embedded in a mixture of EtOH and LR White resin (LR White Embedding Media, Medium catalyzed [Polysciences, Warrington, PA, USA, 17411M-500]) in the following proportions (EtOH: resin): 2:1, 1:1 and 1:2. Each incubation with the mixture was performed at RT and lasted 60 min. Finally, the samples were embedded in pure resin in the following stages: LR White for 15-min stage and LR White overnight. Resin polymerization was performed at 55 °C for 48 h.

#### 2.12.3. Semi-Thin Section Handling for Documentation via TEM with Typing of the Regions of Interest (ROIs) for Immunogold Reactions

The first step involved trimming the LR White blocks with a razor blade to remove excess resin, exposing the surfaces of the samples and obtaining the trapezoidal-shaped blocks. To select the examined tissue area (region of interest, ROI) for transmission electron microscopy (TEM) documentations, the blocks were cut into semi-thin, 600 nm thick sections with a Histo Diamond Knife (Diatome, Nidau, Switzerland) using a Power Tome XL ultramicrotome (RMC, Tucson, AZ, USA). The semi-thin sections were dried on a heating plate (Leica EM MP) and subsequently stained with a dye solution (toluidine blue O, 0.25 g (Sigma-Aldrich) and sodium carbonate anhydrous, Na_2_CO_3_, 0.5 g (Alchem, Poland)). The sections were sealed with the use of the Euparal mounting agent (Roth, Mannheim, Germany). Finally, ultra-thin, 70 nm thick sections were prepared with an Ultra 45° Diamond Knife (Diatome) and collected onto the dull side of nickel grids (200 mesh, Ted Pella (Redding, CA, USA)). 

#### 2.12.4. Colloidal Gold Nanoparticle Immunolocalization

The grids were incubated in 0.02 M glycine (biotechnology grade, Bioshop, catalog number: GLN 001.1) in PBS for 10 min in to quench free aldehyde groups and then gently rinsed in PBS 1 time. Then, to permeabilize the membranes, the grids were incubated with 0.1% Triton X-100 (reagent grade, Bioshop, catalog number: TRX 506.500) diluted in PBS 2 times for 5 min each. Next, the Triton X-100 was rinsed away with PBS 3 times for 5 min. To block nonspecific antigen-binding sites, the grids were placed in 1% bovine serum albumin BSA, albumin fraction V, catalog number: 8076.4, Carl Roth (Mannheim, Germany)) solution diluted in PBS, for 1 h at RT. The BSA was rinsed away with PBS 1 time for 5 min. Afterward, the grids were incubated for 1 h at RT with an anti-TYK-2 primary antibody diluted in 0.1% BSA in PBS (1:25 dilution). Then, the grids were washed in PBS 3 times for 5 min each. Subsequently, a secondary antibody conjugated with colloidal gold particles (Abcam, Cambridge, UK, ab27237, goat anti-rabbit IgG H&L, 20 nm gold, preabsorbed, Lot: GR3200298-7) prepared in 0.1% BSA in PBS (1:10 dilution) was applied, and the grids were incubated for 1 h at RT (in a dark chamber). The following steps involved washing the grids in PBS 3 times for 5 min each, and then in distilled water 3 times for 5 min each. Additionally, the grids were post-fixed for 5 min in 1% glutaraldehyde (Serva Electrophoresis, Heidelberg, Germany) diluted in PBS. The fixative was rinsed away with distilled water 3 times for 5 min each. To improve the contrast, the ultra-thin sections were counterstained with uranyl acetate (10 min) and lead citrate trihydrate (5 min) (Serva). The heavy metal salts were rinsed away 3 times in distilled water. The last step was examination of the grids using a TEM JEM-1011 microscope (Jeol, Tokyo, Japan) at an accelerating voltage of 80 kV. Digital micrographs were collected with the use of an iTEM1233 TEM imaging platform equipped with a Morada camera (Olympus, Münster, Germany) at a magnification ranging from 5 to 20 K.

#### 2.12.5. Digital Quantification of Immunogold Labeled TEM Micrographs

The samples were analyzed with the labeling frequency (LF) method, which involves assessing the number of gold nanoparticles per single cell [14]. Considering the limitations of this approach resulting from the fact that the nanoparticle distribution is influenced by the sizes of the cellular compartments used for the analysis, we selected 10 representative cells per grid with the most similar morphometric parameters. Particular attention was given to cells with a similar length/width ratio as well as nucleus/cytoplasm ratio.

### 2.13. Statistical Analysis

Prism8 software was used to analyze the data between examined test groups (GraphPad (La Jolla, CA, USA), product version: 8.4.2). For each case, the selection of a statistical test was preceded by an assessment of the normality of the distribution. The correlations between the selected parameters were assessed using the Spearman test. Two-tailed *p* values (α < 0.05) were considered to indicate statistical significance. The general discriminative value of each test was determined with the area under the curve (AUC) parameter [15]. ROC curves were generated in the R environment [16] with the pROC package [17]. Patient survival depending on different variables was visualized with Kaplan–Meier curves, and the statistical significance of differences was calculated with the log-rank test [18]. Plots were made in the R environment [15] with the “survival” [19,20] and “survminer” packages [21], and statistical calculations were supported by Statistica 13 (TIBCO Software Inc. (Santa Clara, CA, USA)).

## 3. Results

### 3.1. Immunohistochemical Staining Pattern

**Figure 2:**
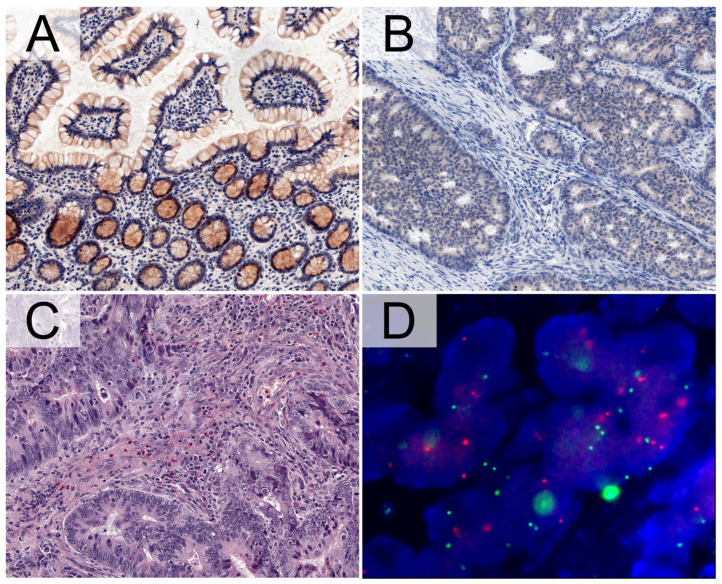
Representative photomicrographs showing TYK2 expression in intestinal glands and goblet cells in normal colonic mucosa (**A**) with adjacent colorectal adenocarcinoma (**B**). The positive immunohistochemical reactions showed higher intensity in healthy surgical margins (**A**) than in cancer (**B**). Hematoxylin and eosin staining revealed the stromal presence of eosinophilic infiltrates visible as oval rounded cells with an acidic cytoplasm forming red rings surrounding bilobed nuclei (**C**). Fluorescence in situ hybridization (FISH) showed positive signals for fluorescent probes against TYK2 (red) and CEP16 (green) in colorectal cancer (**D**). Because the wavelengths of gold and orange are very close, we used the SpectrumOrange filter for TYK2 probe visualization, achieving better digital images. Photomicrographs (**A**–**C**) were taken at 20× magnification, while (**D**) was taken at 63× magnification.

The digital image analysis of immunohistochemical reactions was limited to cancer cells for colorectal adenocarcinomas and to the normal intestinal epithelium of colonic glands in healthy surgical margins. On the basis of an initial verification of IHC staining, significant differences in positive pixel intensities were noted for each TMA core. In adenocarcinomas, digital analysis noted a pronounced distribution of the kinase in the cytoplasm (Figure 2B). Occasionally, positive reactions were also found in the cell membrane. Cytoplasmic and membrane reactivity was also detected in normal intestinal glands, which was consistent with the subcellular localization of TYK2 confirmed in selected proteomic databases [22]. Interestingly, regarding the intestinal glands, pronounced expression of the tyrosine kinase was also detected in goblet cells, which can be seen in the representative photomicrographs (Figure 2A).

### 3.2. TYK2 Expression Decreased in Colorectal Adenocarcinomas

**Figure 3:**
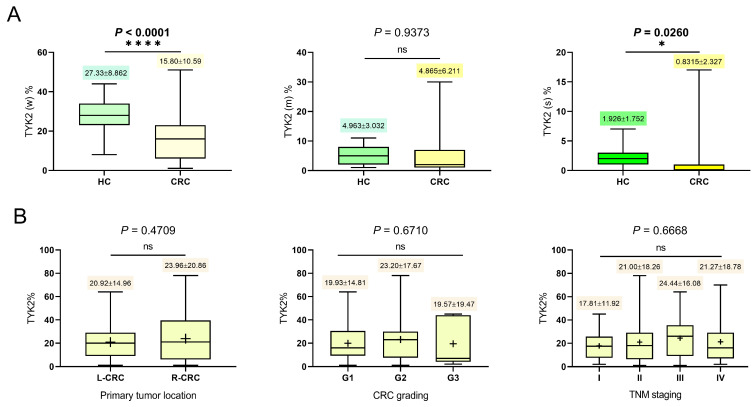
The diagrams show the immunohistochemical expression of TYK2 in normal colon glands designated as healthy control (HC) and colorectal cancer (CRC). The IHC staining reaction was divided and classified into weak (w), moderate (m) and strong (s) intensity considering the individual percentage distribution (TYK2%) of each pixel in the examined core, *p* < 0.05 (*); *p* < 0.0001 (****) (**A**). The selected histopathological CRC data such as tumor location (L-CRC: left-sided, R-CRC: right-sided), tumor grade (G1-G3) and stage (TNM I-IV) were compared with the TYK2% distribution (**B**).

For all positive pixels, there were significant differences in the TYK2 expression level between neoplastic and control tissues; higher levels were observed in normal colonic mucosa (*p* = 0.0004). Even more pronounced differences in immunoreactivity for this kinase were found by paired *t* test for the matched slides (with cancer and surgical margin tissues from the same patients); significantly higher expression of TYK2 was observed in the control tissue (*p* < 0.0001). Regarding the distribution of individual pixels of varying intensity, significant differences were observed for weak (*p* < 0.0001) and strong pixels (*p* = 0.0260), greater percentages of which were found in normal tissue. For moderate pixels, no differences were found between the surgical margins and cancer tissues (*p* = 0.9373). No significant differences were found between the TYK2 pixel IHC response intensity and the CRC clinicopathological data (Figure 3). The percentage of weak pixels among all positive pixels was similar for both cancer cells and normal colonic epithelium; the mean percentage was 82% for CRC as for controls. For moderate pixels, there was a slightly higher mean percentage in the control tissues (15%) than in cancer (14%). The pixels of strongest intensity constituted the lowest mean percentage of all positive pixels, and the percentage was higher in normal colon mucosa (5%) than in adenocarcinomas (2%). Then, TYK2 expression analysis was performed to identify selected histopathological features of clinical importance for colorectal cancer. The TNM, G stage, tumor budding (Bd), tumor size, Crohn-like reaction (CLR) and lympho-vascular invasion (LVI) were taken into consideration. In view of recent reports on the beneficial importance of eosinophils in CRC, the presence of these inflammatory cells was also taken into account in further examination [23,24]. The curves showing the relationships of the kinase and each of the other histopathological features with survival were then independently compared. Taking into account the International Tumor Budding Consensus Conference (ITBCC) 2016 recommendations [25], tumor budding was assessed according to a previously described method [26]. Eosinophil counts (Figure 2C) were assessed only in the tumor stroma in accordance with the guidelines for TIL assessment [27]. Based on the variations in the numbers of eosinophils in the tumor stroma for individual TMA cores, the distribution of these cells was assessed by two independent systems: in the first one, only those cases that contained eosinophilic infiltrates in the stroma (estimated as 1 point) or were completely negative (0 points) were identified. The second semi-quantitative scoring system used included variations in the numbers of these inflammatory cells in the following scoring system: no cells present, 0 points; 1–<10% of cells, 1 point; 10–20% of cells, 2 points; 21–30%, 3 points and >30% eosinophils in the tumor stroma, 4 points. The stromal eosinophilic reaction was assessed for each TMA core via standard H&E staining using the hot spot method. Each case was digitally assessed using CaseViewer software (ŁZ). The obtained results, including the staining pattern, were verified by an experienced pathologist (PD). An independent analysis of the TYK2 level was performed using fluorescent in situ hybridization (FISH) probes (Figure 2D, Table 2). TYK2 expression was significantly higher for cancers not exceeding 2 cm in diameter than for large lesions (*p* = 0.0309, Mann–Whitney U test). With regard to individual pixels, significant differences in tumor size were noted for strong pixels; their percentage was clearly higher in tumors of a smaller diameter than in tumors of a larger size (*p* = 0.0368, Mann–Whitney U test). There were no significant differences between tumor sizes with regard to the distributions of weak (*p* = 0.1563, unpaired *t* test) and moderate pixels (*p* = 0.2473, Mann–Whitney U test). For Bd, there was a negative correlation between tumor budding and the percentage of strong pixels for TYK2 (ρ = −0.270, *p* = 0.0096, Spearman test). There were no significant differences in TYK2 expression according to tumor grade (*p* = 0.6710, ordinary one-way ANOVA) or TNM stage (*p* = 0.6668, ordinary one-way ANOVA). With regard to the clinical stage, only individual T and N parameters were relevant to the expression of this biomarker. For the T parameter, due to the small number of patients with T1 and T4 stages, in further analyses, the patients were divided into two cohorts defined as T1+T2 and T3+T4. For all positive pixels, no significant differences in the TYK2 expression level were found between these two groups (*p* = 0.5627, Mann–Whitney test). Nevertheless, the distribution of strong pixels was clearly higher for the T1+T2 group than for the T3+T4 cohort (*p* = 0.0428, Mann–Whitney test). Interestingly, for the N parameter, a positive correlation was obtained between the number of involved lymph nodes and the expression level of TYK2 for weak pixels (ρ = 0.239, *p* = 0.0242). However, there was no association with TYK2 immunoreactivity between metastatic and nonmetastatic CRC (*p* = 0.8857, Mann–Whitney test). The most common site of distant metastasis was the liver, and eight patients had a single metastasis to this organ. In the other three cases, the cancer spread to the liver and to other parts of the body (the bones, lungs and ovaries). The level of kinase expression was also not dependent on the histological diagnosis. TYK2 expression was not greater in adenocarcinomas of the mucous subtype than in other histological subtypes of CRC (*p* = 0.5704, Mann Whitney test). For ulcerative colonic adenocarcinomas no significant differences in the expression of this tyrosine kinase were found (*p* = 0.9983, Mann–Whitney test). Taking into account the significant influence of the anatomical site on the expression of individual immune factors [26], separate comparative analyses between left-sided (L-CRC) and right-sided (R-CRC) lesions were performed. The classification of CRC to the appropriate location was carried out in accordance with the American Society of Clinical Oncology (ASCO) guidelines [28]. No significant differences in the expression level of TYK2 were found regarding the anatomical site of the primary tumor (*p* = 0.4709, unpaired *t* test). Considering the demographic data, no significant differences in the positive pixel distributions were found to be associated with the sex of the patient (*p* = 0.4406, Mann–Whitney test). TYK2 expression was also not dependent on the age of the examined patient (rho = −0.015, *p* = 0.890, Spearman test).

### 3.3. Higher TYK2 Intensity and Protein Levels in Normal Colonic Mucosa than in Cancer

**Figure 4:**
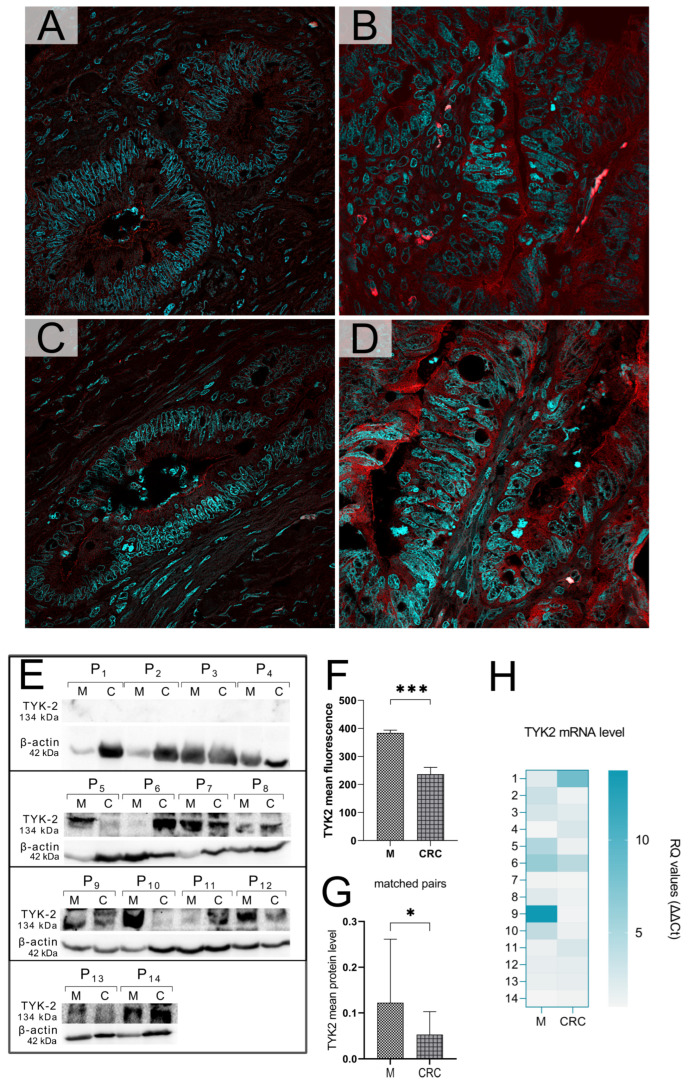
Immunofluorescence staining against TYK2 in colorectal cancer (**A**,**C**) and normal colonic mucosa (**B**,**D**), representing matched pairs secured from two individual patients (**A**–**D**), 60× magnification. Immunoblot analysis showed differences in TYK2 protein levels between healthy surgical margins “M” and adjacent colorectal cancer “CRC” of 14 patients (P1–P14). Bar charts showing significant differences in TYK2 fluorescence intensity (**F**) and protein levels (**G**) between control margins and paired tumors, *p* < 0.05 (*); *p* < 0.001 (***). Comparative analysis of TYK2 mRNA expression levels in paired tissues of individual patients (**H**). Original western blots (WB) are presented in File S1. (**E**). WB bands for TYK2 kinase, B-actin as reference protein; Px refers to the patients and the bottom footnote (x) indicates their individual number, M—healthy surgical margin, C—colorectal adenocarcinomas (**E**).

In order to confirm the IHC results, supporting analyses were implemented, such as immunofluorescence staining against TYK2 and immunoblotting procedures to verify the protein level of the kinase between CRC tissues and healthy surgical margins. IF was performed on whole-glass slides of matched pairs consisting of CRC and adjacent normal colonic mucosa. Fluorescence staining for TYK2 revealed higher TYK2 signal intensities in normal intestinal epithelial cells than in cancer cells (Figure 4A–D,F). Positive fluorescence signals for the kinase were observed on matched slides in both cell membranes and cytoplasm (Figure 4A−D). Semi-quantitative immunoblot analysis revealed that the protein level of TYK2 was significantly higher in healthy controls than in matched colorectal adenocarcinomas (Figure 4E,G).

### 3.4. TYK2 Expression Differs Significantly at the Subcellular Level Between Inflamed Colonic Epithelium and Cancer Cells

**Figure 5:**
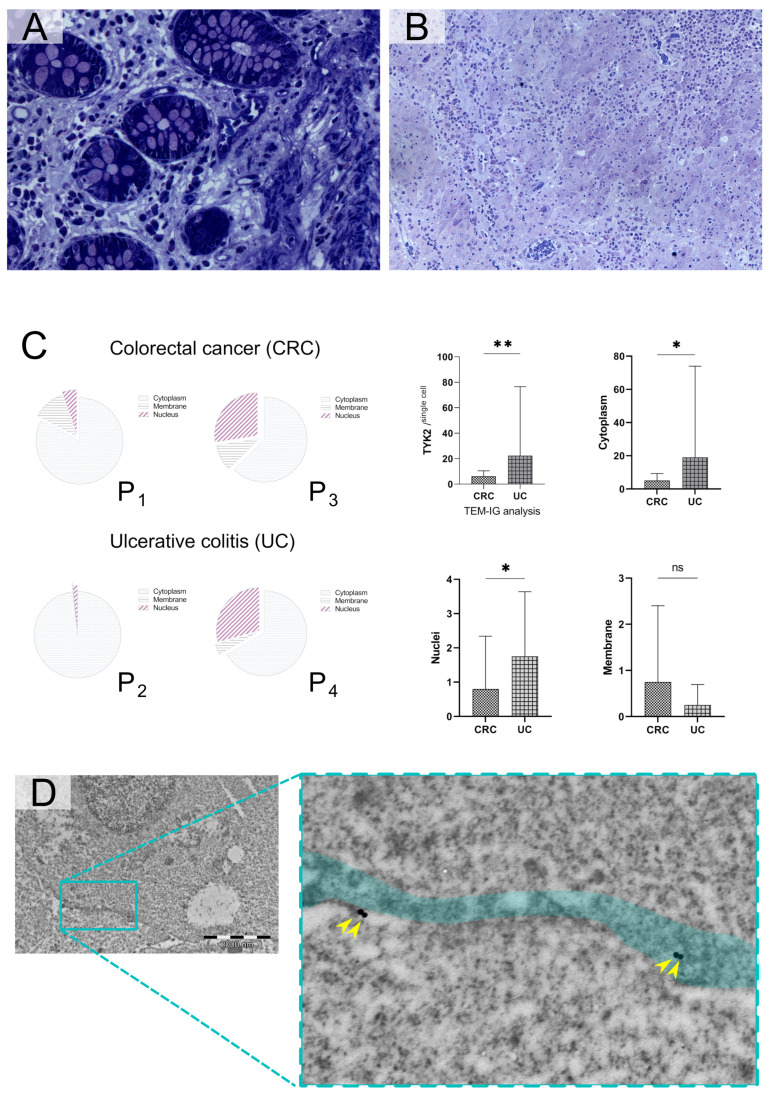
Toluidine blue staining in semi-thin sections showing the microanatomy of ulcerative colitis (**A**), 40× magnification, and colorectal adenocarcinoma (**B**), 25.8× magnification. Graphical presentation of observed differences in TYK2 distribution performed on fresh colonoscopic biopsies from 4 patients (P1–P4) with “de novo” diagnosed ulcerative colitis and colorectal cancer (**C**). Colloidal gold immunolabeling against TYK2 was quantified under TEM imaging in the selected areas of epithelial or cancer cells, *p* < 0.05 (*); *p* < 0.01 (**) (**D**). Representative TEM micrograph showing the specific membrane reaction for TYK2 in the cell membrane (green field, cancer cell). Gold nanoparticles labeled with primary antibodies against TYK2 were considered indicators of positive reactions (yellow arrowheads).

The assessment of TYK2 distribution by TEM considered the cytoplasmic region and cellular membranes. Prior to subcellular analyzes, the regions of interests (ROIs) were properly identified on semi-thin sections from fresh biopsies (Figure 5A,B). Due to the dominant nature of the positive immunohistochemical reactions observed previously, the cell nuclear compartments were excluded from the comparative analysis. The positive points were counted in each area; one point was given for each gold nanoparticle that was present in the ROI. The noticeable differences in the distribution of TYK2 observed between CRC and CU and the nature of the subcellular localization of the antigen encouraged us to perform independent hot spot measurements for each cell. In such measurements, a counting frame was used and fields with a diameter of 7 µm^2^/cell were analyzed. Each compartment of the cell (cytoplasm, nucleus and cell membrane) was assessed independently, and the scores for the cell regions were counted within the microanatomical borders (Figure 5C). The measurements were limited to intestinal epithelial cells or cancer cells only (Figure 5D). For each method, a total of 40 representative cells was assessed (ŁZ, KHL). Additionally, an independent evaluation of the distribution of a positive reaction with the counting of gold nanoparticles was performed. The visualization of TYK2 in individual cell compartments was confirmed for cell organelles such as the mitochondria, endoplasmic reticulum and Golgi apparatus, and the greatest distribution of colloidal gold was found in mitochondria.

### 3.5. TYK2 Gene Methylation and Expression Status

No significant differences in the methylation status (Table 2) or TYK2 mRNA expression level (*p* = 0,3955, paired *t* test, CRC: 3.258 ± 2.343, healthy margins: 4.417 ± 3.417) were found between samples representing paired CRC and healthy intestinal mucosa tissues (Figure 4H). No significant differences were found between UC and CRC fresh biopsies (*p* = 0.3333, Mann–Whitney test, CRC: 1.527 ± 0.01103, UC: 1.043 ± 0.06067).

### 3.6. TYK2 Clinical Importance in Colorectal Adenocarcinoma

**Figure 6:**
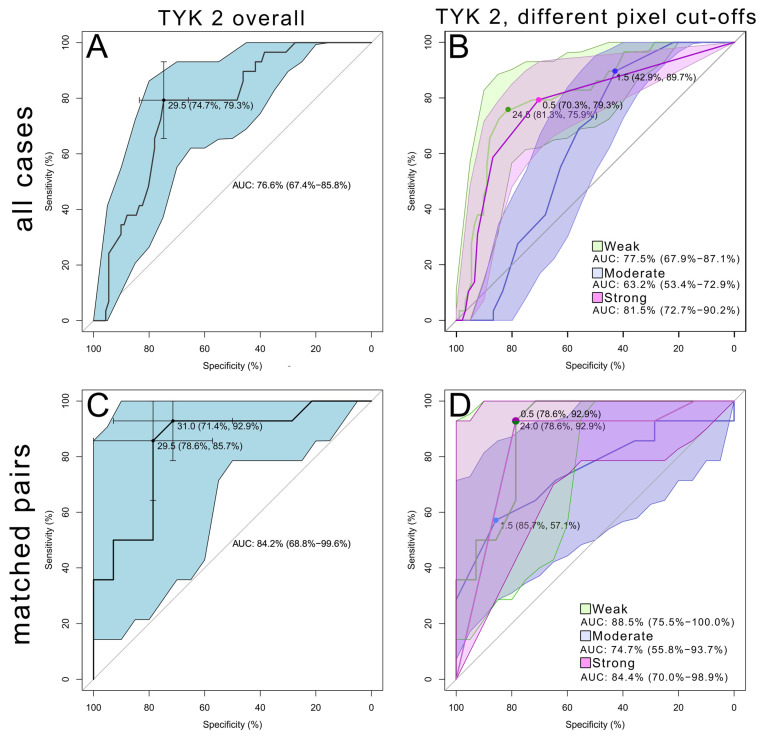
ROC curves showing different cutoffs for TYK2-positive pixels for all patients included in this study (**A**,**B**) and for matched pairs (**C**,**D**). The immunohistochemical staining results, defined as the percentages for all positive pixels (**A**,**C**), were compared with the individual values for weak, moderate and strong pixels for all cases (**B**) and paired specimens (**D**).

To determine the potential predictive value of TYK2 in distinguishing tumor and margin tissue, ROC curves were generated. The ROC curves showed how different cutoffs influenced the specificity ((True Negative)/(True Negative + False Positive)) and sensitivity ((True Positive)/(True Positive + False Negative)) of the test, where tumor tissue was considered a positive outcome. The ROC curves for different variables for the TYK2 pixel strength and the examined cohorts are shown in Figure 6A−D.

**Figure 7:**
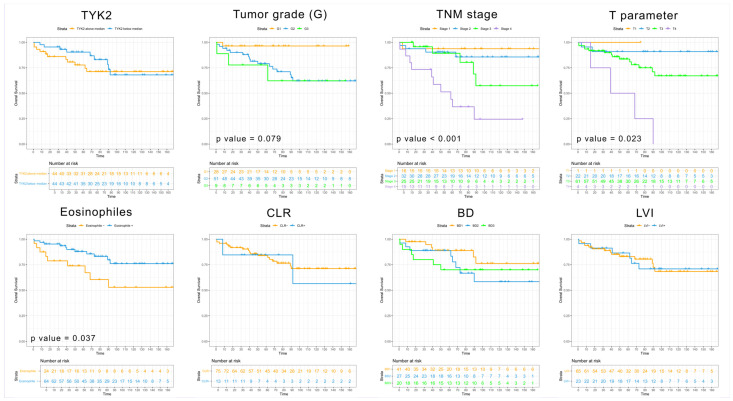
Kaplan–Meier curves visualizing overall survival for colorectal cancer patients (n = 88) depending on different pathologic variables, such as the TYK2 mean, the tumor grade, the TNM stage, the stromal eosinophilic reaction, the Crohn-like reaction (CLR), tumor budding (Bd) and lymphovascular invasion (LVI).

A stromal eosinophilic reaction (SER) in CRC was positively correlated with the percentage of moderately TYK2-positive pixels (ρ = 0.2534, *p* = 0.0172). SER-positive tumors had significantly higher percentages of moderate TYK2 (mean: 5.859%, SD: ±6.896, *p* = 0.0151) and strong TYK2 pixels (mean: 1.094%, SD: ±2.695, *p* = 0.0278) than SER-negative lesions (2.167% ± 2.632 and 0.1250% ± 0.3378, respectively). There was a positive correlation between the percentage of TYK2 moderate pixels and CLR status (ρ = 0.2168, *p* = 0.0424). No significant differences were found in TYK2 expression level between CLR-positive (n = 13) and CLR-negative CRC (n = 75) for all positive pixels (*p* = 0.3211). There was no significant correlation between TYK2-positive pixels and LVI status (ρ = −0.08200, *p* = 0.4475). The percentage of TYK2-positive pixels did not differ between LVI-positive (n = 23) and LVI-negative (n = 65) CRC (*p* = 0.4484). TYK2 expression for all positive pixels was not associated with patient overall survival (*p* = 0.53). Prognostic value was also not shown for LVI (*p* = 0.989), Bd (*p* = 0.25), stages G1–G3 (*p* = 0.07) or a stromal eosinophil score between 0 and 4 points (*p* = 0.269). With regard to TNM staging, the T parameter, tumor grade (G1 vs. G2+G3) and stromal eosinophilic reaction defined as present (+1) or absent (0) were associated with better survival times. Kaplan–Meier curves of patient survival starting from the colectomy procedure to patient death are shown in Figure 7.

## 4. Discussion

Colorectal cancer is the most common cancer associated with inflammatory bowel disease (IBD) [29], and colitis-associated colorectal cancer (CACC) is one of the most serious complications of UC with a worse prognosis than sporadic colonic adenocarcinomas [30]. Therefore, early detection of lesions leading to CRC progression remains crucial. Colonoscopy is a recognized examination that allows for an early screening of CRC [31] and enables the identification and removal of intestinal polyps that may potentially increase the risk of progression to adenocarcinoma [32]. Histopathological examination of colonic biopsies performed to detect histological hallmarks associated with an increased risk of progression toward CRC focuses primarily on the presence and degree of dysplasia; however, this histopathological criterion is not ideal, as currently, there are no clearly established recommendations for handling patients with low-grade dysplasia, and its prevalence remains relatively low in UC patients [33]. Thus, there is a need to detect immune tissue biomarkers associated with neoplastic progression. Nevertheless, no tissue-related proteins have been discovered that have potential diagnostic value as biomarkers for colorectal cancer detection for the validation of endoscopic biopsies [34]. In this study, we found significant differences in TYK2 protein expression between CRC tissues and healthy surgical margins. The negative correlation of the TYK2 expression level with tumor size and Bd may suggest a beneficial role of this tyrosine kinase in the pathogenesis of colorectal cancer. However, we did not confirm the prognostic value of TYK2 in CRC, and it should be noted that this study was conducted on a relatively small cohort of patients. This is one of the limitations of this study. Further research on a wider group of patients will be required to verify the true prognostic significance of this tissue biomarker. Interestingly, we noted a favorable prognostic value for the stromal eosinophilic reaction in cancer. The mere presence of stromal eosinophils was prognostic, but their number in the tumor stroma had no effect on OS. According to the latest reports, tumor eosinophils have a favorable prognostic value in colorectal cancer due to the antitumoral effects of these cells, which are mainly related to the release of IFN-γ [35]. Another limitation was the performance of immunohistochemical staining on histological slides using the tissue microarray (TMA) procedure. To ensure the highest possible fidelity to whole-tissue slides, immunohistochemical reactions were performed on the three most representative TMA cores per patient. Additionally, the results of IHC assessment were supported and verified with independent molecular techniques such as immunofluorescence staining on paired whole-glass slides. IF showed a significantly higher fluorescence intensity for TYK2 in normal mucosal cells than in cancer. Moreover, an independent examination of the TYK2 protein level was performed, which showed a significantly higher level of this kinase in the control samples than in the CRC samples. Immunoblotting was performed on archived frozen tissue specimens corresponding to all paired paraffin blocks included in this study. The conducted analyses confirmed the observed trend for the digital image analysis of immunohistochemical TYK2 expression. Considering the revised Bethesda guidelines for the testing of MSI status [36], our patient cohort did not meet adequate histopathological criteria to support such assessments. The mean age of the patients enrolled in the studies was over 70 years, and the CLR criterion was observed in a small number of cases (14.8%). Based on the fact that colorectal cancer often develops in chronically inflamed colons, an additional assessment of TYK2 distribution was conducted in fresh tissue material obtained endoscopically on previously undiagnosed patients. For that purpose, fresh tissue biopsies collected during colonoscopy from patients with ulcerative colitis and CRC were used to assess the level of TYK2 and its distribution in cells using electron microscopy. Qualitative analyses performed by the TEM-immunogold method revealed the distribution of the kinase TYK2 in the mitochondrial region, both in normal intestinal epithelial cells and in neoplastic cells, with a predominance of mitochondrial localization in patients diagnosed with colitis. Moreover, in the cytoplasm of the examined epithelial cells, a lower level of TYK2 was observed in neoplastic cells than in normal intestinal epithelium. This observation may suggest cancer-specific inactivation of the TYK2 gene, limited translation of this protein during carcinogenesis or the existence of a post-translational mechanism that causes enhanced kinase degradation during the neoplastic process. The lack of association of TYK2 protein expression identified with the performed genetic analyses supports this conclusion. Mozooni et al. observed an increased expression of TYK2 in CRC [37,38], but their analysis only referred to the evaluation of mRNA, not to the protein level or the expression of immunohistochemical reactions. In our patient group, we did not detect significant statistical differences between the mRNA expression levels of control and cancer pairs, although the average gene expression level was lower in cancer than in normal controls. This is consistent with the results of TYK2 expression in IHC reactions, the fluorescence intensity for TYK2 and the TEM analyzes performed. The observed differences could be due to tissue heterogeneity, tumor grade, TNM stages, CRC immune status and the studied patient population, in which geographical location, dietary habits and differences in the composition of gastrointestinal microflora could influence the observed results. CRC, which develops on the basis of chronic inflammation of the colon, can be associated with a number of gene variants that code for selected interleukin receptors and tyrosine kinases. These include *TYK2*, one of the most frequently mutated genes in colitis-associated cancer (CAC), in which missense, splice, synonymous and frameshift mutations have been detected. [39]. Variable expression of the *TYK2* gene can lead to exacerbation of colitis by altering the local microflora of the gastrointestinal tract and hindering the rebuilding of the colonic mucosa of the gastrointestinal tract, as has been observed in *TYK2* deficiency in functional models [10]. 

TYK2 expression is mandatory for the maintenance of mitochondrial respiration in B lymphocytes [40], but thus far, TYK2 has not been shown to be present in mitochondria. We assume that TYK2 in epithelial cells plays an important role in regulating cellular oxidation and that the reduced mitochondrial kinase distribution in CRC may result in decreased ATP production. In BRAF-mutated CRC and polyps, affected oxidation and impaired mitochondrial outer membrane (MOM) permeability have been found [41]. The TEM results obtained in this study may suggest an important biological role of TYK2 in the mechanism towards colorectal adenocarcinoma carcinogenesis, since ulcerative colitis is often a pre-cancerous state for this cancer. Another limitation of our electron microscopy analysis was the small number of samples that were examined in this study. To compensate for the small number of cases, TEM analysis was performed on the 10 most representative cells for each patient. Although the ultrastructural analyses of the kinase yielded interesting data, elucidation of the precise role of TYK2 in the pathogenesis of CRC requires additional functional tests in both in vitro and in vivo models. Another limitation in this study was the restriction of the analyses to primary colorectal adenocarcinomas. Our analyses did not allow us to deduce whether the expression level of TYK2 allows for the differential diagnosis of adenocarcinomas of extraintestinal origin. There were no significant differences between metastatic CRC (mCRC) and nonmetastatic colonic lesions. Performing this type of research would allow us to determine whether TYK2, as an inflammatory marker, shows tissue specificity for tumors of a similar histopathological subtype, but of varied etiology and primary anatomical location (arising from different organs). In this study, we showed the decreased expression of TYK2 at the protein level in neoplastic cells compared with normal intestinal epithelium and epithelial cells from inflamed colons, which underlines the potential value of TYK2 in monitoring CRC progression. Considering that early diagnosis is key in CRC therapy, our results support the use of TYK2 in the assessment of colonic biopsies to monitor the risk of progression to adenocarcinoma, and CACC in particular. The immunohistochemical results for TYK2 expression may support the controlled evaluation of colonic biopsies and thus the use of TYK2 immunohistochemistry in early CRC screening. We have also shown that digital analysis of IHC reactions allows broader conclusions to be drawn than semi-quantitative analyses. We found significant differences in the strength of individual pixels between the corresponding clinical and pathological data. The detection of such dependencies would not have been possible with semi-quantitative scoring systems.

## 5. Conclusions

Our results support the legitimacy of implementing digital analyses in routine practice in cancer pathology. In human adenocarcinomas, TYK2 kinase was significantly decreased at the protein level, which was confirmed by several independent methods. This result underlines the importance of this kinase in the carcinogenesis of CRC. Further research should include comparative analyses of adenocarcinomas with polyps and assess blood or plasma TYK2 levels to define the overall potential of this tyrosine kinase for use in the early detection of CRC.

## Figures and Tables

**Table 1:** Patient clinicopathological characteristics.

Variable	Archived FFPE Blocks		
Specimen Characteristic	Colorectal Cancer	Normal Colonic Mucosa	Cancer-Associated Normal Colonic Mucosa
Age (years), mean [range]	70.07 [42–88]	69.44 [50–88]	69.93 [52–86]
*N*			
Patient cohort	88	27	14
Sex (female/male)	44/44	12/15	6/8
pTNM			
1	16		
2	32		
3	25		
4	15		
pT			
(T_1_ + T_2_)	24		
(T_3_ + T_4_)	64		
pN			
N_0_	56		
N_1_	22		
N_2_	10		
pM			
mCRC	15		
non-mCRC	73		
Grading			
G_1_	28		
G_2_	52		
G_3_	8		
Anatomical site			
Left-sided CRC	55		
Right-sided CRC	29		
Histology			
mucinous	12		
non-mucinous	76		
Macroanatomical subtype			
ulcerated	39		
non-ulcerated	49		
Tumor size			
≥2 cm	74		
<2 cm	14		

**Table 2:** Genetic analyses carried out on FFPE blocks paired with colorectal cancer (CRC) and ad-jacent normal colonic mucosa.

TYK2 Promoter Region DNA Methylation Level N = 14 Pairs of Tissue, 28 Specimens							
Number		Localization of the Analyzed CG Dinucleotides (GRCh38/hg38)		Mean Methylation Level in Tumor Samples [%]	Range of Methylation Level in Tumor Samples [%]	Mean Methylation level in Control Samples [%]	Range of Methylation Level in Control Samples [%]
1		chr19:10,380,345		2.7	1.5–6,3	2.2	1.1–4.5
2		chr19:10,380,349		3.3	1.8–8	4.2	1.8–10.2
3		chr19:10,380,355		5.3	1.9–26.5	4	2.2–10.6
4		chr19:10,380,358		2.3	1–4.4	3.2	1.3–10.2
Fluorescence in situ hybridization (FISH) N = 6 pairs of whole-glass slides, 12 cases							
**Histology**							
**Colonic Adenocarcinomas**					**Normal Colonic Mucosa**		
**Patient Number**	**TYK2 (Gold)**		**16cen (Green)**	**TYK2/16cen**	**TYK2 (Gold)**	**16cen (Green)**	**TYK2/16cen**
1	192		221	0.87	165	211	0.78
2	178		148	1.20	195	162	1.20
3	161		175	0.92	130	143	0.91
4	250		259	0.97	182	174	1.05
5	149		152	0.98	163	143	1.14
6	227		169	1.34	189	196	0.96

## Data Availability

The data underlying this article will be shared on reasonable request to the corresponding author.

## Supplementary Materials

The following supporting information can be downloaded at: https://www.mdpi.com/article/10.3390/cancers16213665/s1, File S1: Original western blots.
