# Supplementary figures and images for: TYK2 Protein Expression and Its Potential as a Tissue-Based Biomarker for the Diagnosis of Colorectal Cancer
Source: Cancers (Basel). 2024 Oct 30;16(21):3665. doi: 10.3390/cancers16213665 (PMC11545102; doi:10.3390/cancers16213665)

TYK-2: 134 kDa

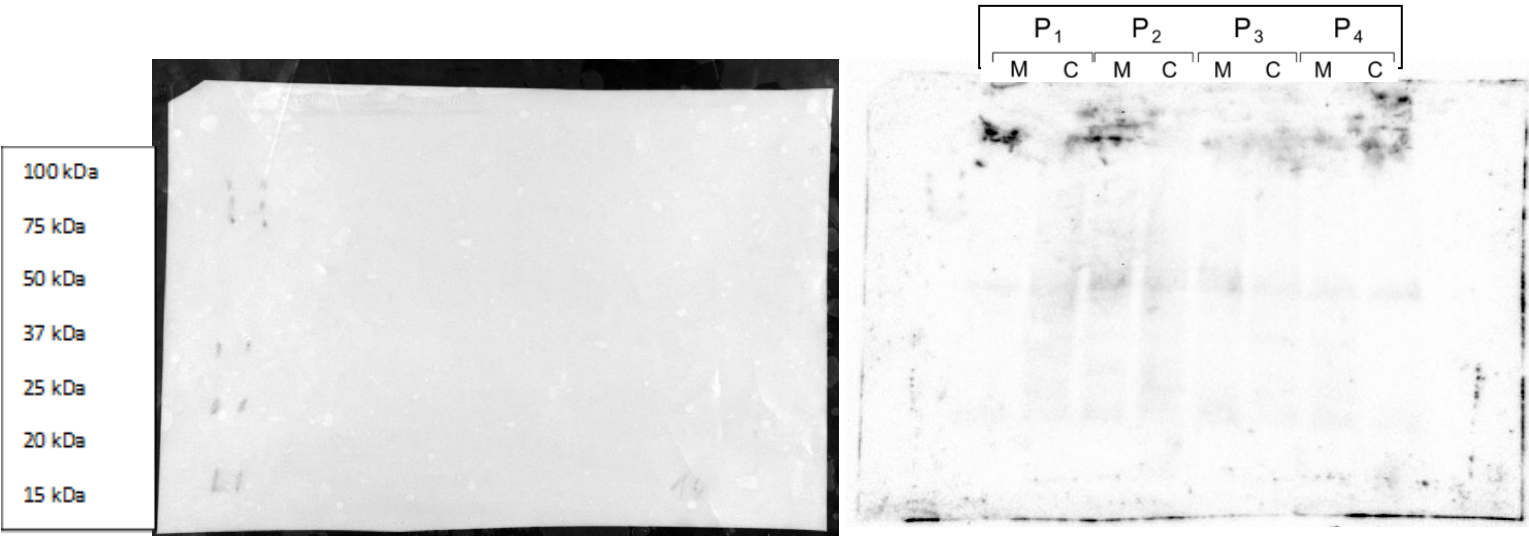

$\beta$ -actin: 40 kDa

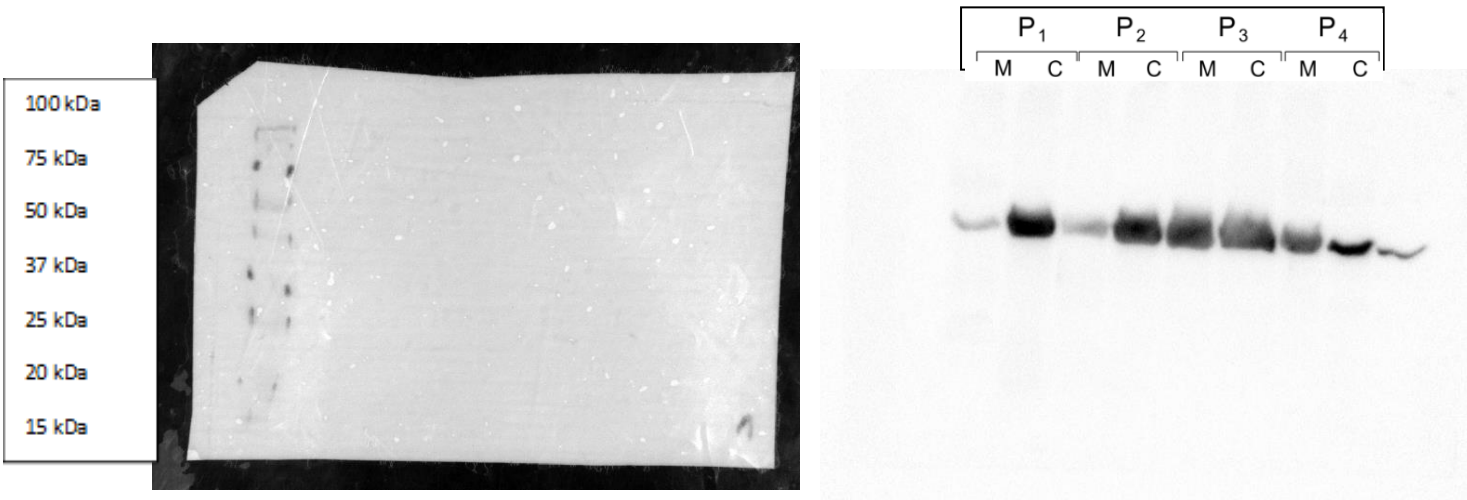

**TYK-2: 134 kDa**

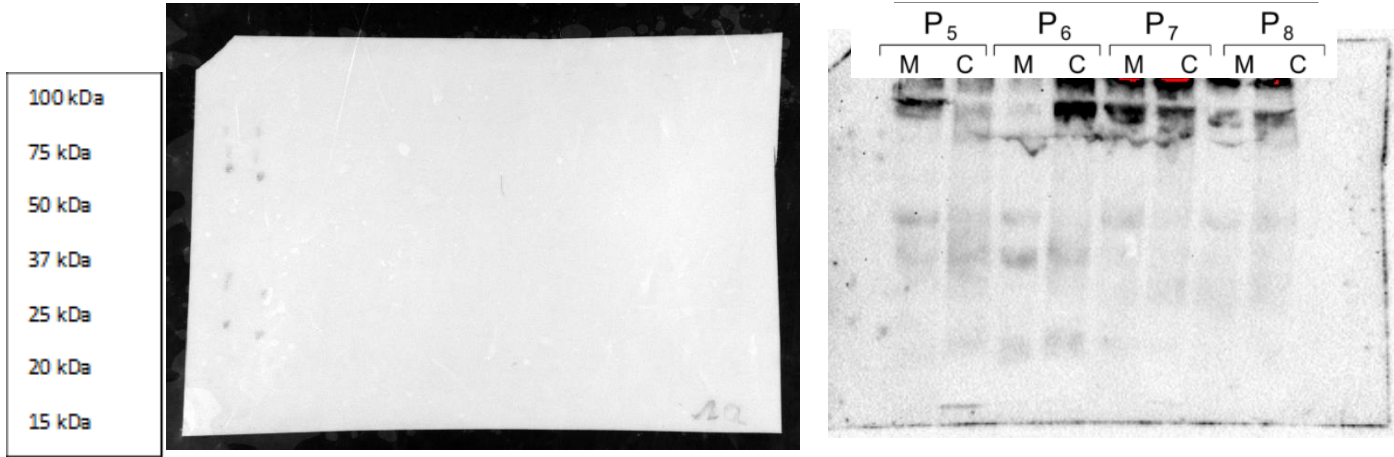

**$\beta$ -actin: 40 kDa**

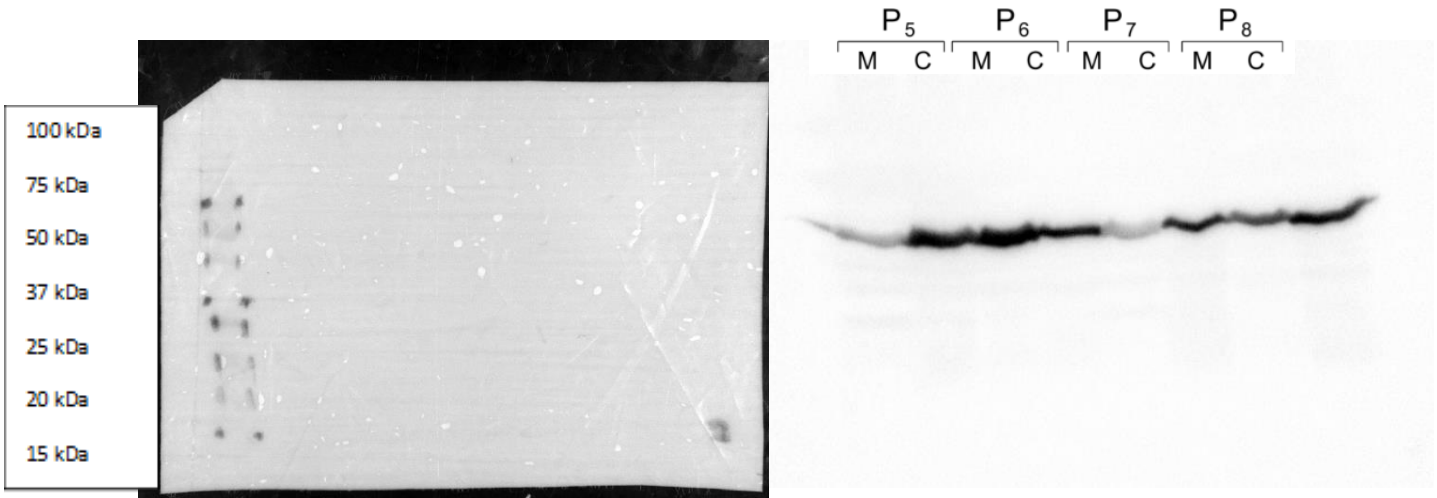

TYK-2: 134 kDa

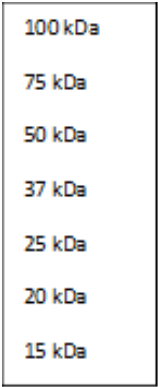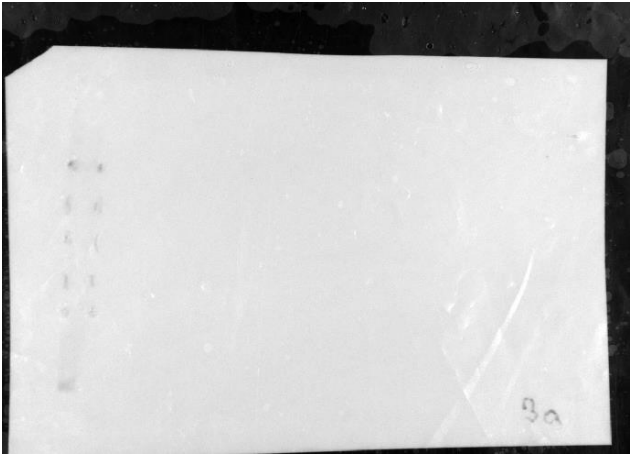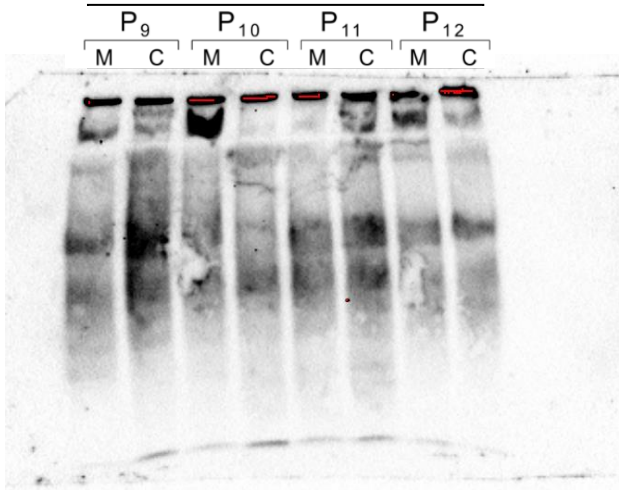

$\beta$ -actin: 40 kDa

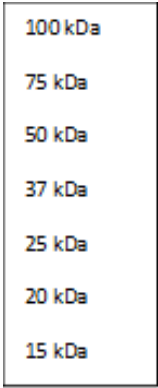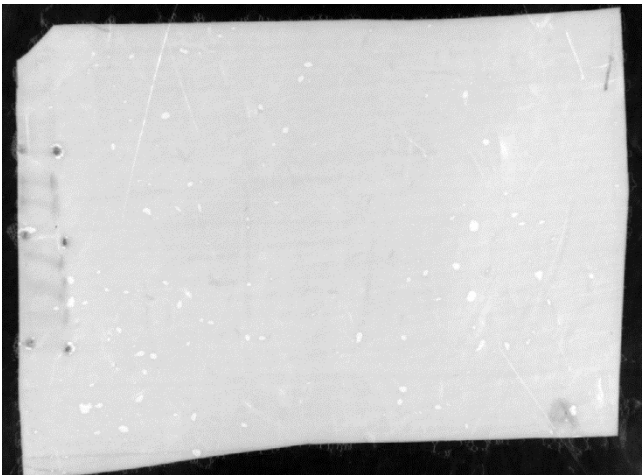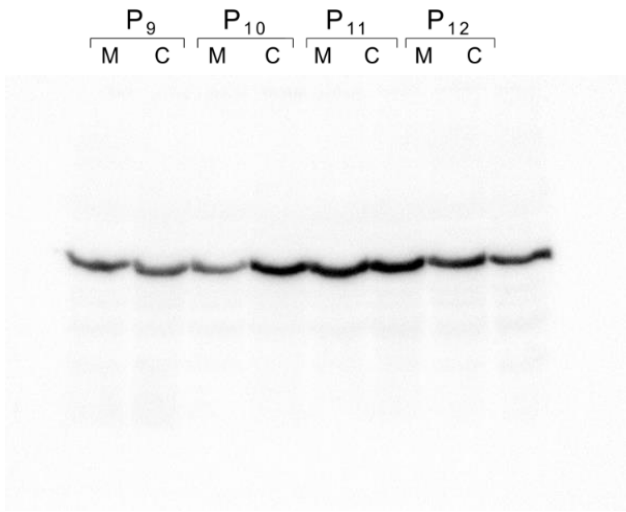

TYK-2: 134 kDa

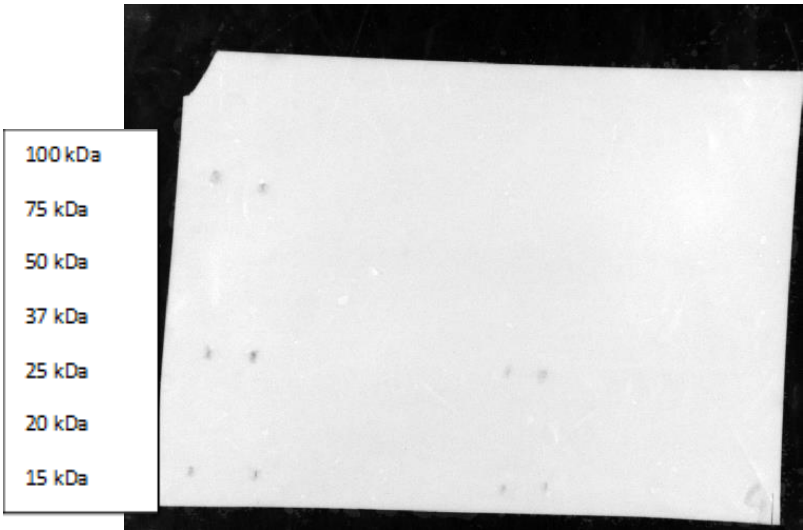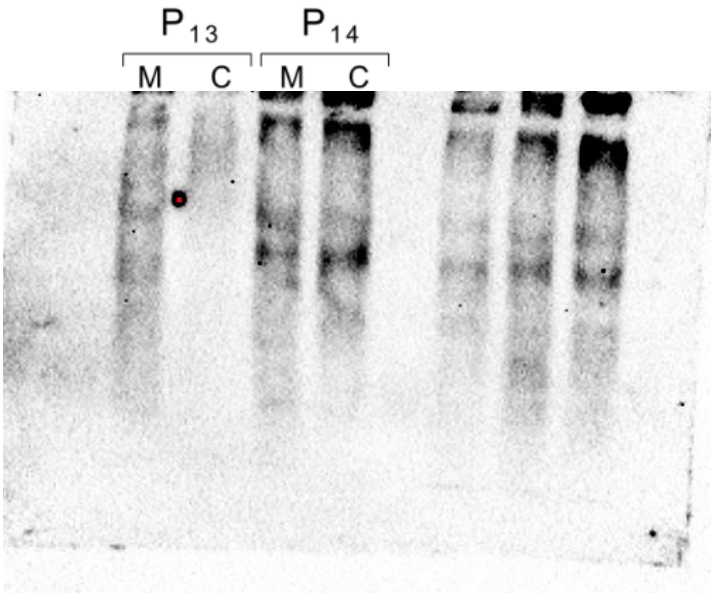

$\beta$ -actin: 40 kDa

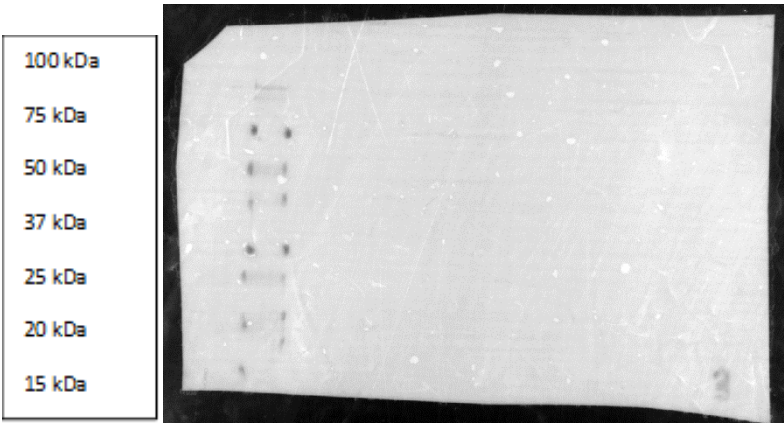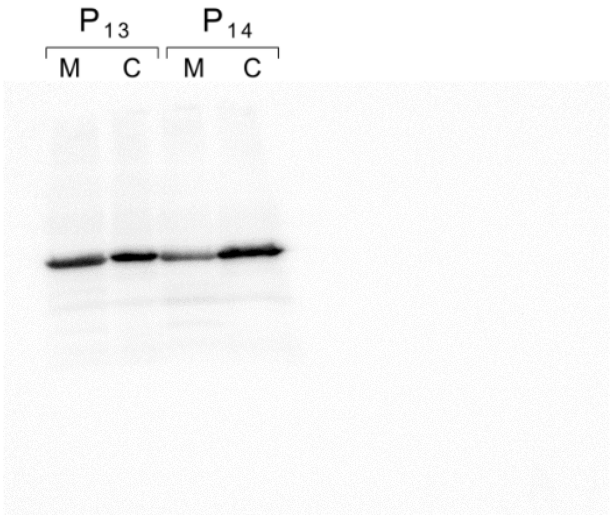

Supplement: Supplementary file 1 [file cancers-16-03665-s001.zip › cancers-3246991-File S1.pdf]
